# Something New—Nitric Acid a Remedy for Stings and Poisons

**Published:** 1882-01-20

**Authors:** A. L. Barry

**Affiliations:** Ringgold, Georgia


### SOMETHING NEW—NITRIC ACID A REMEDY FOR STINGS AND POISONS.

*Editors of The Southern Medical Record—*

GENTLEMEN: I must give you an adventure which I had with a hive of bees, and the results, which, by a grand mistake, in using nitric acid instead of sweet oil, I made the discovery that the acid was a veritable specific for the sting of bees. Being cleanly shaved and unprotected, I undertook to transfer a hive of bees which becoming enraged covered my face and hands by the thousand. After fighting my best for a short time, I with difficulty made my way into the house and called for sweet oil. Being in great pain, and even alarmed at the possible result, without time, and in no condition for reflection as to the best means to be used for relief, I held my hand in a cupped position and directed it to be filled with sweet oil, but in the haste and confusion they snatched up a bottle of nitric acid and poured my hand full, which I instantly applied, first to my forehead and then over my entire face and hands, when my grand-daughter exclaimed, "Oh! grandpa, stop—stop, it is the wrong bottle, it is nitric acid!' Alarmed with a new danger, I bethought me of soda, to nutralize the acid, and called for it, but before it arrived I was most agreeably amazed and delighted to find the pain of the stings suddenly relieved as if by magic, and to find that no cauterizing effect or injury had been sustained, but that the poison was gone, and even the swelling rapidly subsided.

A few weeks after the above singular discovery, Mr. D. came to me with hands inflamed and much swollen from the effects of poison oak. I at once thought of the bee remedy and applied nitric acid; at first cautiously on the back of one hand, and, seeing no pain or cauterizing effect, I applied it to the other, and to the entire part inflamed, with speedy and absolute relief to the poisonous symptoms.

A. L. BARRY, M. D.

*Ringgold, Georgia, December,* 1881.

[The statements contained in the above note from Dr. B. are certainly remarkable, and if borne out by future experiments, must prove a singular and valuable discovery, and will illustrate how little is yet known of the therapeutic properties of even our most familiar drugs. We hope that this matter will be further tested by our correspondent, and by others in the profession, and that the facts will be sent us for publication.—ED.]

